# Uropathogenic *Escherichia coli* in Iran: Serogroup distributions, virulence factors and antimicrobial resistance properties

**DOI:** 10.1186/1476-0711-12-8

**Published:** 2013-04-29

**Authors:** Hassan Momtaz, Azam Karimian, Mahboobeh Madani, Farhad Safarpoor Dehkordi, Reza Ranjbar, Meysam Sarshar, Negar Souod

**Affiliations:** 1 Department of Microbiology, ShahreKord Branch, Islamic Azad University, P.O. Box: 166, ShahreKord, Iran; 2 Department of Microbiology, Falavarjan Branch, Islamic Azad University, Isfahan, Iran; 3 Graduated of Veterinary Medicine, ShahreKord Branch, Islamic Azad University, ShahreKord, Iran; 4 Molecular Biology Research Center, Baqiyatallah University of Medical Sciences, Tehran, Iran; 5 Young Researchers club, Jahrom Branch, Islamic Azad University, Jahrom, Iran

**Keywords:** *Escherichia coli*, Urinary tract infections, Serogroups, Virulence factors, Antibiotic resistance

## Abstract

**Background:**

Urinary tract infections (UTIs) are one of the most common bacterial infections with global expansion. These infections are predominantly caused by uropathogenic *Escherichia coli* (UPEC).

**Methods:**

Totally, 123 strains of *Escherichia coli* isolated from UTIs patients, using bacterial culture method were subjected to polymerase chain reactions for detection of various O- serogroups, some urovirulence factors, antibiotic resistance genes and resistance to 13 different antibiotics.

**Results:**

According to data, the distribution of O1, O2, O6, O7 and O16 serogroups were 2.43%, besides O22, O75 and O83 serogroups were 1.62%. Furthermore, the distribution of O4, O8, O15, O21 and O25 serogroups were 5.69%, 3.25%, 21.13%, 4.06% and 26.01%, respectively. Overall, the *fim* virulence gene had the highest (86.17%) while the *usp* virulence gene had the lowest distributions of virulence genes in UPEC strains isolated from UTIs patients. The *vat* and *sen* virulence genes were not detected in any UPEC strains. Totally, *aadA1* (52.84%), and *qnr* (46.34%) were the most prevalent antibiotic resistance genes while the distribution of *cat1* (15.44%), *cmlA* (15.44%) and *dfrA1* (21.95%) were the least. Resistance to penicillin (100%) and tetracycline (73.98%) had the highest while resistance to nitrofurantoin (5.69%) and trimethoprim (16.26%) had the lowest frequencies.

**Conclusions:**

This study indicated that the UPEC strains which harbored the high numbers of virulence and antibiotic resistance genes had the high ability to cause diseases that are resistant to most antibiotics. In the current situation, it seems that the administration of penicillin and tetracycline for the treatment of UTIs is vain.

## Background

Urinary tract infections (UTIs) are one of the most frequent infectious diseases around the world. Urinary tract infections comprise ranges of disorders including pyelonephritis (infection of the kidney) and cystitis (infection of the bladder), which are defined by the presence of microorganisms in urinary tract [1]. Foxman (2003) indicated that 40%-50% of women have UTIs throughout their lives [2]. Also, previous report of WHO showed that the UTIs are common causes of febrile illness in 3–8% of girls and 1% of boys [3].

*Escherichia coli* (E. coli) is the most important cause of UTI [4,5]. Uropathogenic *E. coli* (UPEC) strains have shown certain virulent properties, including iron uptake systems, adhesins, specific O: K: H serotypes and synthesis of cytotoxins. All of these properties contribute to colonization and invasion of the bacterium [6]. The successful colonization of UPEC in the urinary tract depends on the expression of fimbrial adhesion proteins, which facilitate attachment of the bacterium to the urothelium, and on the presence of specific bacterial genes that encode virulence factors [7-9].

The *Escherichia coli* strains are normally identified by serological typing of their H (flagellar), O (lipopolysaccharide) and in some cases, K (capsular) surface antigens. Overall, 174 O-serogroups are described for *E. coli*[10]. The O-serogroups of UPEC strains are related to certain virulence factor profile of each strain. Previous studies reported that O1, O2, O4, O6, O7, O8, O15, O16, O18, O21, O22, O25, O75 and O83 serogroups are preferentially associated with UPEC strains [11-18].

Some of the most important virulence genes of UPEC strains which are associated with severe UTIs are aerobactin (*aer*), P fimbriae (*pap*), type 1 fimbriae, afimbrial adhesin I (*afa*I), hemolysin (*hly*), cytotoxic necrotizing factor 1 (*cnf* 1), aerobactin (*aer*), S fimbriae (*sfa*), adhesins and fimbriae [19,20]; however, other virulence genes such as *kpsMT*, *ompT*, *usp*, *iroN*, *iha*, *set 1*, *astA*, group II capsule synthesis; *sfa*/*foc*, S and *F1C* fimbriae; *iutA*, *traT*, serum resistance; and *fimH*, are known to be involved in pathogenicity of this organism [21-23].

The treatment of diseases caused by this bacterium often requires antimicrobial therapy; however, antibiotic-resistant strains of bacteria cause more severe diseases for longer periods of time than their antibiotic-susceptible counterparts. Several studies showed that antibiotic resistance in UPEC is increasing nowadays [24,25]. Because of the high antimicrobial resistances of UPEC strains in Portugal (26%), Italy (25%) and Spain (21%) [26], the identification of bacterial resistance genes seems to be essential to reduce the treatment costs. To our knowledge, the epidemiology and prevalence of serogroups, virulence factors and antimicrobial resistance properties of UPEC strains isolated from patients with UTIs are unknown in Iran. So, the current survey was carried out in order to determine the serogroups, virulence factors and antimicrobial resistance properties of UPEC strains isolated from patients with UTIs in Iran.

## Methods

### Bacterial strains

A total of 123 *E. coli* strains isolated from patients with symptomatic UTIs were enrolled in the current investigation. The patients were hospitalized or visited the emergence room at Baqiyatallah Hospital in Tehran, Iran. The strains were isolated from pure cultures and identified in the laboratory of Molecular Biology Research Center at Hospital. The strains which were biochemically confirmed as *E. coli*- positive, were kept in Luria-Bertani /glycerol at −70°C.

### DNA isolation

Bacterial strains were subcultured overnight in Luria-Bertani broth (Merck, Germany) and genomic DNA was extracted from typical colonies of *E. coli* using DNA extraction kit (DNP™, CinnaGen, Iran) according to manufacturer’s instruction.

### *Detection of* uropathogenic *E. coli serogroups, virulence factors and antibiotic resistance genes*

In the present study various PCR assays were used for detection of serogroups, virulence factors and antimicrobial resistance genes of UPEC *E. coli isolates*. Table 1 showes the primers applied for detection of UPEC virulence genes, Table 2 indicates the primers used for detection of UPEC serogroups and finally in Table 3 the primers used for detection of antimicrobial resistant genes in uropathogenic *E. coli* are shown. The amplified products were visualized by ethidium bromide staining after gel electrophoresis of 10 μL of the final reaction mixture in 1.5% agarose.

**Table 1:** **Primers used for detection of virulence genes in uropathogenic *Escherichia coli***

**Gene**	**Primer name**	**Primer sequence (5'-3')**	**Size of product (bp)**	**Reference**
***set-1***	set-1-F	GTGAACCTGCTGCCGATATC	147	[21]
	set-1-R	ATTTGTGGATAAAAATGACG		
***sen***	Sen-F	ATGTGCCTGCTATTATTTAT	799	[21]
	Sen-R	CATAATAATAAGCGGTCAGC		
***astA***	astA-F	ATGCCATCAACACAGTATAT	110	[21]
	astA-R	GCGAGTGACGGCTTTGTAGT		
***sigA***	sigA-F	TCCTCGGTATTATTTTATCC	408	[21]
	sigA-R	CGTAACCCCTGTTGTTTCCAC		
***sap***	Sap_f	TACCCTCCACAACAGAGAATG	832	[21]
	Sap-R	TACCCTCCACAACAGAGAATG		
***pic***	Pic-F	ACTGGATCTTAAGGCTCAGGAT	500	[21]
	Pic-R	GACTTAATGTCACTGTTCAGCG		
***pap***	pap3	GCAACAGCAACGCTGGTTGCATCAT	336	[27]
	pap4	AGAGAGAGCCACTCTTATACGGACA		
***cnf1***	cnf1	AAGATGGAGTTTCCTATGCAGGAG	498	[27]
	cnf2	TGGAGTTTCCTATGCAGGAG		
***hlyA***	hly1	AACAAGGATAAGCACTGTTCTGGCT	1177	[27]
	hly2	ACCATATAAGCGGTCATTCCCGTCA		
***sfa***	sfa1	CTCCGGAGAACTGGGTGCATCTTAC	410	[28]
	sfa2	CGGAGGAGTAATTACAAACCTGGCA		
***afa***	afa1	GCTGGGCAGCAAACTGATAACTCTC	750	[28]
	afa2	CATCAAGCTGTTTGTTCGTCCGCCG		
***iuc***	iuc1	ATGAGAATCATTATTGACATAATTG	1482	[29]
	iuc2	CTCACGGGTGAAAATATTTT		
***fim***	fim1	GAGAAGAGGTTTGATTTAACTTATTG	559	[30]
	fim2	AGAGCCGCTGTAGAACTGAGG		
***papGI***	papGJ96-F	TCGTGCTGAGGTCCGGAATTT	461	[31]
	papGJ96-R	TGGCATCCCCCAACATTATCG		
***papGII***	papGIA2-F	GGGATGAGCGGGCCTTTGAT	190	[31]
	papGIA2-R	CGGGCCCCCAAGTAACTCG		
***papGIII***	prsJ96-F	GGCCTGCAATGGATTTACCTGG	258	[31]
	prsJ96-R	CCACCAAATGACCATGCCAGAC		
***kpsMT***	kpsM481F	CCATCGATACGATCATTGCACG	400	[32]
	kpsM481R	ATTGCAAGGTAGTTCAGACTCA		
***iha***	IHA-F	CTGGCGGAGGCTCTGAGATCA	827	[23]
	IHA-R	TCCTTAAGCTCCCGCGGCTGA		
***iron***	IRONEC-F	AAGTCAAAGCAGGGGTTGCCCG	665	[23]
	IRONEC-R	GACGCCGACATTAAGACGCAG		
***ompT***	ompT-F	ATCTAGCCGAAGAAGGAGGC	559	[33]
	ompT-R	CCCGGGTCATAGTGTTCATC		
***usp***	usp *-F*	ACATTCACGGCAAGCCTCAG	440	[22]
	usp *-R*	AGCGAGTTCCTGGTGAAAGC		
***iss***	Iss-F	ATCACATAGGATTCTGCCG	309	[34]
	Iss-R	CAGCGGAGTATAGATGCCA		
***irp2***	Irp2-F	AAGGATTCGCTGTTACCGGAC	413	[34]
	Irp2-R	AACTCCTGATACAGGTGGC		
***tsh***	Tsh-F	ACTATTCTCTGCAGGAAGTC	824	[34]
	Tsh-R	CTTCCGATGTTCTGAACGT		
***vat***	Vat-F	TCCTGGGACATAATGGTCAG	981	[34]
	Vat-R	GTGTCAGAACGGAATTGT		
***cva***	Cva-F	TGGTAGAATGTGCCAGAGCAAG	1181	[34]
	Cva-R	GAGCTGTTTGTAGCGAAGCC		

**Table 2:** **Primers used for detection of uropathogenic *Escherichia coli *serogroups**[35]

**Serogroup**	**Target gene**	**Primer name**	**Primer sequence (5’-3’)**	**Size of product (bp)**
**O1**	*wzx*	wl-14632	GTGAGCAAAAGTGAAATAAGGAACG	1098
		wl-14633	CGCTGATACGAATACCATCCTAC	
**O6**	*wzy*	wl-14646	GGATGACGATGTGATTTTGGCTAAC	783
		wl-14647	TCTGGGTTTGCTGTGTATGAGGC	
**O7**	*wzx*	wl-14648	CTATCAAAATACCTCTGCTGGAATC	610
		wl-14649	TGGCTTCGAGATTAAACCTATTCCT	
**O8**	*orf469*	wl-14652	CCAGAGGCATAATCAGAAATAACAG	448
		wl-14653	GCAGAGTTAGTCAACAAAAGGTCAG	
**O16**	*wzx*	wl-14654	GGTTTCAATCTCACAGCAACTCAG	302
		wl-14655	GTTAGAGGGATAATAGCCAAGCGG	
**O21**	*wzx*	wl-14676	CTGCTGATGTCGCTATTATTGCTG	209
		wl-14677	TGAAAAAAAGGGAAACAGAAGAGCC	
**O75**	*wzy*	wl-17413	GAGATATACATGGGGAGGTAGGCT	511
		wl-17414	ACCCGATAATCATATTCTTCCCAAC	
**O2**	*wzy*	wl-14636	AGTGAGTTACTTTTTAGCGATGGAC	770
		wl-14637	AGTTTAGTATGCCCCTGACTTTGAA	
**O4**	*wzx*	wl-14642	TTGTTGCGATAATGTGCATGTTCC	664
		wl-14643	AATAATTTGCTATACCCACACCCTC	
**O15**	*wzy*	wl-14672	TCTTGTTAGAGTCATTGGTGTATCG	183
		wl-14673	ATAAAACGAGCAAGCACCACACC	
**O18**	*wzx*	wl-14656	GTTCGGTGGTTGGATTACAGTTAG	551
		wl-14657	CTACTATCATCCTCACTGACCACG	
**O22**	*wzx*	wl-14660	TTCATTGTCGCCACTACTTTCCG	468
		wl-14661	GAAACAGCCCATGACATTACTACG	
**O25**	*wzy*	wl-14666	AGAGATCCGTCTTTTATTTGTTCGC	230
		wl-14667	GTTCTGGATACCTAACGCAATACCC	
**O83**	*wzx*	wl-14668	GTACACCAGGCAAACCTCGAAAG	362
		wl-14669	TTCTGTAAGCTAATGAATAGGCACC	
***E. coli***	*16SrRNA*	wl-3110	AGAGTTTGATCMTGGCTCAG	919
		wl-3111	CCGTCAATTCATTTGAGTTT	

**Table 3:** **Primers used for detection of antimicrobial resistant genes in uropathogenic *Escherichia coli***

**Antimicrobial agent**	**Resistance gene**	**Sequence**	**Size (bp)**	**References**
**Streptomycin**	*aadA1*	(F) TATCCAGCTAAGCGCGAACT	447	[36]
		(R) ATTTGCCGACTACCTTGGTC		
**Gentamicin**	*aac(3)-IV*	(F) CTTCAGGATGGCAAGTTGGT	286	[36]
		(R) TCATCTCGTTCTCCGCTCAT		
**Sulfonamide**	*sul1*	(F) TTCGGCATTCTGAATCTCAC	822	[36]
		(R) ATGATCTAACCCTCGGTCTC		
**Beta-lactams**	*blaSHV*	(F) TCGCCTGTGTATTATCTCCC	768	[36]
		(R) CGCAGATAAATCACCACAATG		
**Beta-lactams**	*CITM*	(F) TGGCCAGAACTGACAGGCAAA	462	[36]
		(R) TTTCTCCTGAACGTGGCTGGC		
**Chloramphenicol**	*cat1*	(F) AGTTGCTCAATGTACCTATAACC	547	[36]
		(R) TTGTAATTCATTAAGCATTCTGCC		
**Chloramphenicol**	*cmlA*	(F) CCGCCACGGTGTTGTTGTTATC	698	[36]
		(R) CACCTTGCCTGCCCATCATTAG		
**Tetracycline**	*tet(A)*	(F) GGTTCACTCGAACGACGTCA	577	[37]
		(R) CTGTCCGACAAGTTGCATGA		
**Tetracycline**	*tet(B)*	(F) CCTCAGCTTCTCAACGCGTG	634	[37]
		(R) GCACCTTGCTGATGACTCTT		
**Trimethoprim**	*dfrA1*	(F) GGAGTGCCAAAGGTGAACAGC	367	[38]
		(R) GAGGCGAAGTCTTGGGTAAAAAC		
**Quinolones**	*qnr*	(F) GGGTATGGATATTATTGATAAAG	670	[39]
		(R) CTAATCCGGCAGCACTATTTA		

### Antimicrobial susceptibility testing

Antimicrobial susceptibility tests was performed by the Kirby–Bauer disc diffusion method using Mueller–Hinton agar (HiMedia Laboratories, Mumbai, India, MV1084), according to the Clinical and Laboratory Standards Institute guidelines [40]. After incubating the inoculated plates aerobically at 37°C for 18–24 h in an aerobic atmosphere, the susceptibility of the *E. coli* isolates to each antimicrobial agent was measured and the results were interpreted in accordance with interpretive criteria provided by CLSI (2006). *E. coli* ATCC 25922 was used as quality control organisms in antimicrobial susceptibility determination.

### Statistical analysis

The data were analyzed using *SPSS* software (Version 17.SPSS Inc, United States) to find any significant correlation between incidences of virulence factors and antibiotics resistance genes of uropathogenic *E. coli* serogroups isolated from patients with urinary tract infection. Statistical significance was regarded at a *P* value < 0.05.

### Ethical issues

In the current study we tried to protect the life, health, dignity, integrity, rights to self-determination, privacy, and confidentiality of personal information of research subjects. We conform to generally accepted scientific principles, be based on a thorough knowledge of the scientific literature, other relevant sources of information, and adequate laboratory and, as appropriate, animal experimentation. All samples were taken from volunteer patients for this research. All ethical issues were considered and this research was performed with hospitals’ permission. The name and characters, personal information and even patients’ illnesses and their medical information remained secret. In addition, in this cooperation agreement we stated that this research will help urology and microbiology and is able to clarify the epidemiology and prevalence of stereotypes, virulence factors and antimicrobial resistance of UPEC strains isolated from patients with urinary tract infection. All of the patients showed their satisfaction in order to use their sample in this investigation especially to determine antibiotic resistance in UPEC strains.

## Results and discussion

Our results revealed high distribution of UPEC serogroups isolated from patients with urinary tract infection (Table 4). Totally, O25 (26.01%), O15 (21.13%) and O16 (10.56%) had the highest while O18 (0.81%), O75 (1.62%), O22 (1.62%) and O83 (1.62%) had the lowest distributions of UPEC serogroups isolated from patients with UTIs (Table 4). Besides, the serogroups of 13.82% UPEC strains isolated from these patients could not be detected and were diagnosed as non-detected serogroups (Table 4).

**Table 4:** **Distribution of virulence genes in uropathogenic *Escherichia coli *serogroups isolated from urinary tract infections in Iran**

**Gene**	**O1 (3)**	**O6 (13)**	**O7 (3)**	**O8 (4)**	**O16 (3)**	**O21 (5)**	**O75 (2)**	**O2 (3)**	**O4 (7)**	**O15 (26)**	**O18** **(1)**	**O22 (2)**	**O25 (32)**	**O83 (2)**	**Non detect (17)**
***set1 *(98)**	3	12	3	4	3	3	-	1	4	25	-	2	28	-	10
***astA *(26)**	-	3	1	1	-	1	-	-	-	8	-	-	10	-	2
***sigA *(26)**	1	5	-	-	1	-	1	1	1	-	1	1	11	1	2
***sap *(32)**	-	-	-	3	2	1	-	-	1	10	1	-	14	-	-
***pic *(16)**	2	8	1	-	-	-	1	-	-	4	-	-	-	-	-
***sfa *(66)**	1	8	1	-	3	4	1	2	5	10	1	-	23	-	7
***afa *(10)**	-	-	-	-	-	-	1	-	-	3	-	-	4	-	2
***cnf1 *(62)**	1	4	-	2	1	4	-	-	4	15	1	-	30	-	-
***hlyA *(62)**	-	8	2	-	-	2	-	2	4	21	-	1	9	1	10
***iuc *(13)**	-	-	-	-	-	-	-	-	-	3	-	-	10	-	-
***fim *(106)**	3	10	3	3	1	5	2	3	7	23	1	2	30	2	11
***kspMT *(5)**	-	1	-	1	-	-	-	1	-	2	-	-	-	-	
***ompT *(6)**	-	-	-	-	-	-	-	1	-	1	-	-	3	-	1
***usp *(2)**	-	-	-	-	-	-	-	-	-	1	-	-	-	-	1
***iss *(10)**	-	2	-	-	-	1	-	-	2	3	-	-	2	-	-
***irp2 *(14)**	-	-	-	-	1	-	-	-	-	1	-	-	10	-	1
***vat *(12)**	1	-	1	2	-	-	1	2	-	-	1	-	3	1	-
***cva *(6)**	-	6	-	-	-	-	-	-	-	-	-	-	-	-	-
***pap *(62)**	1	3	1	2	1	3	1	1	4	20	-	1	20	1	3
***papGI *(10)**	-	1	1	-	-	1	-	-	-	4	-	1	-	1	1
***papGII *(19)**	-	-	-	-	-	-	1	1	4	3	-	-	8	-	2
***papGIII *(62)**	1	2	1	2	1	2	1	1	2	19	-	-	19	-	11
***iha *(22)**	-	2	-	1	-	1	-	-	-	3	1	1	10	1	2
***iron (52)***	-	2	1	1	3	1	-	1	2	10	1	-	21	2	7

Overall, *fim* and *set1* had the highest distributions of virulence genes while *usp*, *kpsMT*, *cva* and *ompT* had the lowest (Table 4). As it was shown in Table 4, we were not able to identify the distributions of *sen* and *tsh* virulence genes of UPEC in our population. Table 5 shows the distributions of antibiotic resistance genes of UPES isolated from patients with UTIs. It was recognized that *aadA1* (52.84%) and *qnr* (46.34%) had the highest while *cat1* (15.44%) and *cmlA* (15.44%) had the lowest distributions of antibiotic resistance genes (Table 5). Also, the distributions of *tetA*, *tet B*, *dfrA1*, *dfrA1*, *aac(3)-IV*, *sul1*, *blaSHV* and *CITM* antibiotic resistance genes were 43.80%, 36.58%, 21.95%, 22.76%, 36.58%, 27.64% and 39.83%, respectively (Table 5).

**Table 5:** **Distribution of antimicrobial resistance genes in uropathogenic *Escherichia coli *serogroups isolated from urinary tract infections in Iran**

**UPEC Serogroup**	**Antibiotic resistance genes**										
	***aadA1***	***tetA***	***tetB***	***dfrA1***	***qnr***	***aac(3)-IV***	***sul1***	***blaSHV***	***CITM***	***cat1***	***cmlA***
**O1 (3)**	1	1	1	1	1	1	1	1	1	-	-
**O6 (13)**	6	6	3	4	5	1	11	7	7	1	1
**O7 (3)**	1	-	2	2	2	1	2	-	2	-	-
**O8 (4)**	1	2	-	2	-	1	1	1	2	-	-
**O16 (3)**	2	1	1	1	-	1	1	1	3	1	-
**O21 (5)**	-	3	2	1	2	2	1	1	-	1	1
**O75 (2)**	1	1	1	1	-	1	-	2	1	-	1
**O2 (3)**	1	2	1	1	1	1	2	2	-	2	-
**O4 (7)**	1	4	2	-	6	4	-	3	2	2	1
**O15 (26)**	8	10	9	11	6	10	10	4	10	-	1
**O18 (1)**	-	-	1	1	1	2	1	-	-	1	-
**O22 (2)**	1	1	-	1	-	1	1	2	1	-	-
**O25 (32)**	31	18	14	1	28	1	1	1	17	10	14
**O83 (2)**	1	-	-	-	3	1	2	2	2	-	-
**Non detected (17)**	10	4	8	-	2	-	11	7	1	1	-
**Total (123)**	65 (52.84%)	53 (43.8%)	45 (36.58%)	27 (21.95%)	57 (46.34%)	28 (22.76%)	45 (36.58%)	34 (27.64%)	49 (39.83%)	19 (15.44%)	19 (15.44%)

The disk diffusion method indicated that the UPEC serogroups had maximum resistance to penicillin (100%) and tetracycline (73.98%) antibiotics while resistance to nitrofurantoin (5.69%) was minimum (Table 6). Besides, the UPEC serogroups had 53.65%, 25.20%, 30.89%, 33.33%, 29.26%, 20.32% and 36.58% resistances to streptomycin, chloramphenicol, sulfamethoxazol, enrofloxacin, enrofloxacin, lincomycin, cephalothin and ampicillin antibiotics, respectively. Totally, resistance to gentamycin, ciprofloxacin and trimethoprim were minimal (17.07%, 19.51% and 16.26%), respectively (Table 6).

**Table 6:** **Antimicrobial resistance properties in uropathogenic *Escherichia coli *serogroups isolated from urinary tract infections in Iran**

**UPEC Serogroup**	**P10 (%)**	**TE30 (%)**	**S10 (%)**	**C30 (%)**	**SXT (%)**	**GM10 (%)**	**NFX5 (%)**	**L2 (%)**	**CF30 (%)**	**CIP5 (%)**	**TMP5 (%)**	**F/M300 (%)**	**AM10 (%)**
**O1 (3)**	3	2	2	-	1	-	-	1	-	-	1	-	1
**O6 (13)**	13	8	6	2	10	1	2	10	6	5	4	2	6
**O7 (3)**	3	-	2	-	2	1	2	1	-	-	1	-	2
**O8 (4)**	4	2	2	-	1	1	-	1	-	-	1	-	2
**O16 (3)**	3	2	1	-	-	-	-	1	-	-	1	-	2
**O21 (5)**	5	4	2	1	1	1	1	1	-	2	-	1	-
**O75** **(2)**	2	2	-	1	-	-	-	-	1	-	-	-	1
**O2 (3)**	3	3	-	2	1	-	-	-	1	-	-	1	-
**O4** **(7)**	7	6	5	2	-	4	1	2	3	6	-	-	2
**O15 (26)**	26	19	14	-	10	10	6	15	4	5	10	1	10
**O18 (1)**	1	-	-	1	-	1	-	-	-	-	1	-	-
**O22 (2)**	2	1	-	-	1	1	-	-	2	-	1	-	1
**O25 (32)**	32	30	18	22	-	1	27	4	-	6	-	-	17
**O83 (2)**	2	-	-	-	1	-	2	-	1	-	-	-	1
**Non detected (17)**	17	12	14	-	10	-	-	-	7	-	-	2	-
**Total (123)**	123 (100%)	91 (73.98%)	66 (53.65%)	31 (25.20%)	38 (30.89%)	21 (17.07%)	41 (33.33%)	36 (29.26%)	25 (20.32%)	24 (19.51%)	20 (16.26%)	7 (5.69%)	45 (36.58%)

In this table P10 = penicillin (10 u/disk); TE30 = tetracycline (30 μg/disk); S10 = streptomycin (10 μg/disk); C30 = chloramphenicol (30 μg/disk); SXT = sulfamethoxazol (25 μg/disk); GM10 = gentamycin (10 μg/disk); NFX5 = enrofloxacin (5 μg/disk); L2 = lincomycin (2 μg/disk); CF30 = cephalothin (30 μg/disk); CIP5 = ciprofloxacin (5 μg/disk); TMP5 = trimethoprim (5 μg/disk); F/M300 = nitrofurantoin (300 μg/disk); AM10 = ampicillin (10 u/disk).

Our results revealed that the UPEC strains were able to be one of the major causative agents of UTIs in Iran and this finding was in accordance with the previous study which mentioned that 150 million people are diagnosed as UTI- positive annually [41]. Also, The UPEC strains are isolated from the uterine contents in the majority (82–100%) of clinical pyometra cases [42,43]. Therefore, such a high prevalence of serogroups, virulence factors and antibiotic resistance genes in UPEC strains exhibits that there is a high risk of developing incurable diseases.

Several investigations have been performed on the prevalence of UPEC strains in UTI cases in Iran [44,45]. Kalantar et al. [44] showed that the *E. coli* was the most frequent occurring pathogen (54.80%) in patients with severe UTIs. Esmaeili [45] demonstrated that the *E. coli* was the most common cause of UTI in human. Also, previous study has been reported that the incidence of UTI has been increased recently [46].

Another study in Iran showed that 140 out of 244 patients with UTIs (57.37%) had the high levels of *E. coli* infection which was lower than our percentage (66.12%) [47]. Ghorashi et al. [48] declared that 77% of patients with UTIs were positive for *E. coli* which was higher than our results. Several investigations have been performed on the prevalence of UPEC strains in UTI cases around the world including Brazil [49], United States [50], Europe, and Canada [51].

Our results indicated that there were several serogroups of *E. coli* in UTI positive patients. There was statistical significant differences between the presence of O25 and O18 serogroups (*P* < 0.01), O25 and O83, O75, O22, O7, O2 and O1 (*P* < 0.05). Totally, O25 and O15 were the most prevalent serogroups. Since 1980 [52], many investigators reported that several O-serogroups were found with variable frequencies in UTIs patients. Similar results have been reported recently too [12,53]. The previous survey showed that the majority of uropathogenic *E. coli* strains such as O4, O6, O14, O22, O75 and O83 were *HlyA* + *CNF1*+ and expressed P-fimbriae or MRHA type III, whereas O18 serogroup strains were *HlyA* + *CNF1*− and P-fimbriated [14] which was in accordance with those of us.

Based on our results, there were significant differences about (*P* < 0.01) between the presence of *fim, tsh* and *sen* virulence genes as well as set1, *tsh* and *sen* genes (*P* < 0.05) and also between *fim* and *usp* genes of isolated *E. coli* strains (*P* < 0.05). Therefore, *fim* and *set1* were the most common virulence genes. Arabi et al. [54] showed the similar results of UPEC virulence genes in Iran. Arabi et al. [54] indicated that the *fim* and *sfa* fimbriae genes were observed in 92.7% of isolates, separately. Also, Asadi Karam et al. [55] showed that the *fim* genes were the most prevalent virulence genes of UPES strains. Karimian et al. [56] proved that *fimH* gene with the frequency rate of 79.67% was the most and *tsh* and *usp* genes with the frequency rate of 0.0% and 1.62% respectively were the least common virulence genes in *E. coli* strains isolated from patients with urinary tract infections. Also, Karimian et al. [56] showed that the presence of *cnf1, hlyA, pap, iroN, afa, iuc, iha, ompT* and *irp2* virulence genes were 50.4, 50.4, 50.4, 42.27, 8.13, 10.56, 17.88, 4.87 and 11.38%, respectively. Other virulence genes of UPEC strains like *aatA*, *aggR* and *stbA*[47] and *stx1* and *stx2*[57] have been reported from Iran previously. Another investigation announced that the prevalence rate of *fimH*, *fyuA*, *kpsMTII* and *iucD* genes were above 75% likewise *papC*, *papG*, *sat*, *iron*, *usp* and *traT* were between 35-65% [12].

Our results contrary to other studies indicated that there are the possibilities of the existence of multiple virulence genes in UPEC strains isolated from UTIs patients [58,59]. The importance of UPEC strains’ *sfa* gene in patients with severe UTIs has been reported previously [59,60] while Abe et al. [11] and Santo et al. [59] reported lower prevalence of *sfa* gene among UPEC. Another study indicated that *usp* and *iha* virulence genes were present in 63.7% and 34.1% of all *E. coli* isolates [22] which both were higher than our findings.

The statistical analyses were significant among *aadA1, cat1* and *cmlA* antibiotic resistance genes (*P* < 0.05). This correlation was demonstrated among tetracycline and nitrofuration (*P* < 0.05) and also penicillin, trimethoprim and gentamycin (*P* < 0.05) too. High frequency of resistant UPEC strains to one or more antimicrobials was observed in the present work and it was in agreement with previous studies [61,62].

The most common antibacterial drugs in UTIs’ treatment are trimethoprim-sulfamethoxazole, cephalosporins, semi-synthetic penicillins with or without beta-lactamase inhibitors and quinolones [62,63]; however, our results proved that resistance to penicillin, sulfamethoxazole, trimethoprim and cephalotin were 100%, 30.89%, 16.26% and 20.32%, respectively. Oliveira et al. from Brazil [61] reported that 90% of UPEC strains possessed at least one of the resistant genes, the prevalence of them were as follows: *traT* (76%), *aer* (41%), *PAI* (32%), *sfa* (26%), *pap* (25%), *cnf1* (18%), *afa* (6%), and *hly* (5%) and the most common were ampicillin (51%) and trimethoprim-sulfamethoxazole (44%). According to Idrees Muhammad et al. [64] results, there were high prevalence of class 1 integrons (43.56%), sulfamethoxazole resistance genes *sul1* (45.54%) and *sul2* (51.48%) as well as quinolone resistance genes in multi drug resistance UPEC isolates in Pakistan. Farshad et al. [65] showed the high prevalence of resistance genes to ampicillin (80.2%), co-trimoxazole (76%) and tetracycline (70.8%) in Iran.

Recently, trimethoprim-sulfamethoxazole was used as a standard antibiotic for a calculated UTIs therapy and due to the increased resistance of UPEC strains to this class of antibiotics, fluoroquinolones as broad-spectrum antimicrobial agents have been used with increasing frequency in complicated as well as uncomplicated UTIs [66], but after a short time resistance to fluoroquinolones was emerged [67]. Previous study showed that more than 10% of the *E. coli* isolated in 2000–2002 from intensive care unit patients in European and North American were resistant to ciprofloxacin [68], while this amount of resistance was increased significantly in our evaluation.

Gulsun et al. [69] reported that the sensitivity of the UPEC strains to the norfloxacin, ciprofloxacin, netilmicin, amikacin, ceftriaxone, gentamicin, nitrofurantoin, amoxicillin-clavulanate, trimethoprim/sulfamethoxazole and ampicillin were 89%, 85%, 80%, 78%, 74%, 72%, 71%, 58%, 45%, 35%, respectively which was somewhat similar to our progeny. In a study carried out in India, the highest resistance have been shown against amoxicillin (67.3%) and least against nitrofurantoin (57.3%) [70] which was similar to our findings. On the other hand our results revealed that in the current situations the nitrofurantoin is a choice drug due its lowest antibiotic resistance in UPEC strains. Eighty five to ninety two percent of UPEC strains were sensitive to nitrofurantoin in previous study [53].

Our results showed that resistance to chloramphenicol was 25.5% but chloramphenicole is a forbidden antibiotic and the high antibiotic resistance to this drug in our study indicated the irregular and unauthorized use of this drug in medicine treatments. Unfortunately, not only in medicine, but also veterinarians in many fields of veterinary such as large animal internal medicine, poultry and even aquaculture, use this antibiotic as a basic one. Therefore, in a very short period of time, antibiotic resistance will appear.

## Conclusions

Based on our results, O25 serotype, *fim* virulence gene, *aadA1* antibiotic resistance gene and finally resistance to penicillin had highest frequencies in UPEC strains isolated from UTIs patients. The results of our study revealed the high presence of UPEC strains in patients with UTIs in Iran. To our knowledge, our study is the first report of direct detection of serogroups, virulence factors and antimicrobial resistance properties of uropathogenic *E. coli* strains in Iran. Due to the high prescription of antibiotics in humans and even animals, antibiotics resistance has been increased in UPEC strains and we recommend using antibiotics only in severe conditions and applying strong antibiotics and multi antibiotic descriptions.

## Competing interests

The authors declare that they have no competing interests.

## Authors’ contributions

HM carried out the molecular genetic studies, participated in the primers sequence alignment and drafted the manuscript. AK and MS carried out the sampling and culture method. RR participated in the primers sequence alignment. FSD, MM and NS participated in the design of the study, performed the statistical analysis and writing the manuscript. All authors read and approved the final manuscript.
